# An evolutionary and physiological perspective on cell-substrate adhesion machinery for cell migration

**DOI:** 10.3389/fcell.2022.943606

**Published:** 2022-08-25

**Authors:** Julio C. Fierro Morales, Qian Xue, Minna Roh-Johnson

**Affiliations:** Department of Biochemistry, University of Utah, Salt Lake City, UT, United States

**Keywords:** focal adhesion, cell migration, cell-substrate adhesion, evolution, *in vivo* environment

## Abstract

Cell-substrate adhesion is a critical aspect of many forms of cell migration. Cell adhesion to an extracellular matrix (ECM) generates traction forces necessary for efficient migration. One of the most well-studied structures cells use to adhere to the ECM is focal adhesions, which are composed of a multilayered protein complex physically linking the ECM to the intracellular actin cytoskeleton. Much of our understanding of focal adhesions, however, is primarily derived from *in vitro* studies in Metazoan systems. Though these studies provide a valuable foundation to the cell-substrate adhesion field, the evolution of cell-substrate adhesion machinery across evolutionary space and the role of focal adhesions *in vivo* are largely understudied within the field. Furthering investigation in these areas is necessary to bolster our understanding of the role cell-substrate adhesion machinery across Eukaryotes plays during cell migration in physiological contexts such as cancer and pathogenesis. In this review, we review studies of cell-substrate adhesion machinery in organisms evolutionary distant from Metazoa and cover the current understanding and ongoing work on how focal adhesions function in single and collective cell migration in an *in vivo* environment, with an emphasis on work that directly visualizes cell-substrate adhesions. Finally, we discuss nuances that ought to be considered moving forward and the importance of future investigation in these emerging fields for application in other fields pertinent to adhesion-based processes.

## Introduction

Cell-substrate adhesion is pivotal for a variety of key cellular processes (Khalili and Ahmad, 2015). In Metazoan systems, cell adhesion to a surrounding extracellular matrix (ECM) substrate initiates the generation of mechanosensitive cues, which are transduced into biochemical signals used by cells to promote efficient migration through their microenvironment (Lock et al., 2008; Geiger et al., 2009; De Pascalis and Etienne-Manneville, 2017). One of the most well-studied structures cells use to adhere to the ECM is focal adhesions, nanostructures composed of a multilayered protein complex physically linking the ECM to the intracellular actin cytoskeleton (Lo, 2006; Kanchanawong et al., 2010; Case and Waterman, 2015). Briefly, integrin heterodimers–which act as transmembrane receptors–bind to the ECM and initiate formation of the intracellular protein complex. Studies utilizing super-resolution microscopy have shown that this complex is composed of three distinct functional layers: an integrin signaling layer, a force transduction layer and an actin regulatory layer (Kanchanawong et al., 2010; Case and Waterman, 2015). Focal adhesions are highly dynamic, assembling at the front/leading edge of the cell, and disassembling at the trailing edge, allowing for efficient cell migration. Dynamics such as assembly rate, adhesion lifetime, and the kinetics of individual proteins components are also spatially and temporally controlled by environmental factors and intrinsic signals such as calcium, which has been identified as an important regulator controlling focal adhesion turnover *via* calpain mediated degrading and disassembling of focal adhesion molecules (Bhatt et al., 2002; Wei et al., 2012; Chang et al., 2017)*.* Furthermore, these dynamics are often used in research as proxies of the entire focal adhesion structure and function during processes such as migration.

**FIGURE 1:**
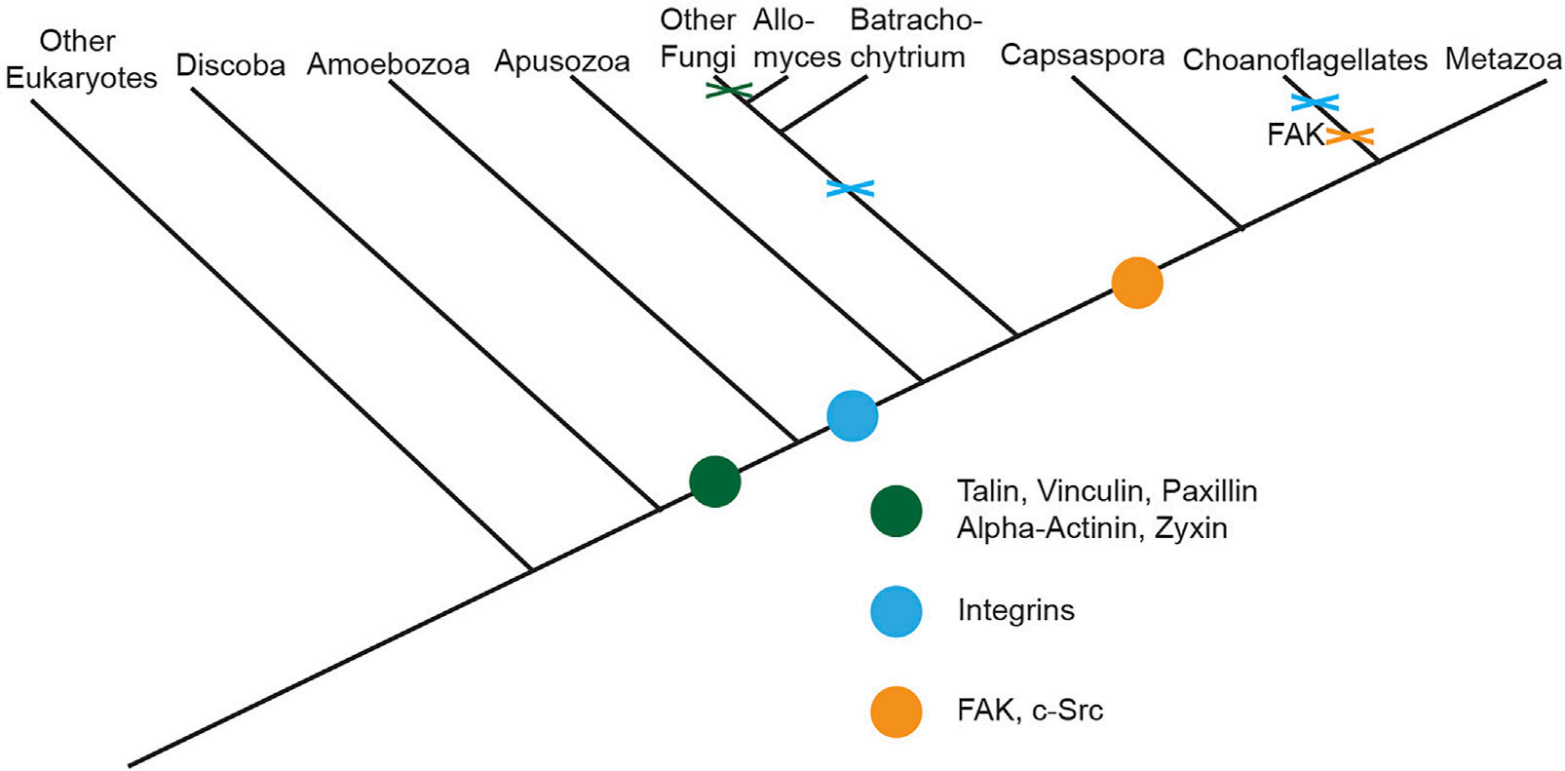
Evolution of cell-substrate adhesion machinery across Eukaryotes. Cladogram depicting the origin and lineage-specific losses of known core components of Metazoan cell-substrate adhesion machinery across Eukaryotes. Origin of a component is indicated by a colored dot along the cladogram while loss of components prior to the divergence of a lineage is denoted *via* a colored X. Figure is adapted from (Sebe-Pedros et al., 2010).

**FIGURE 2:**
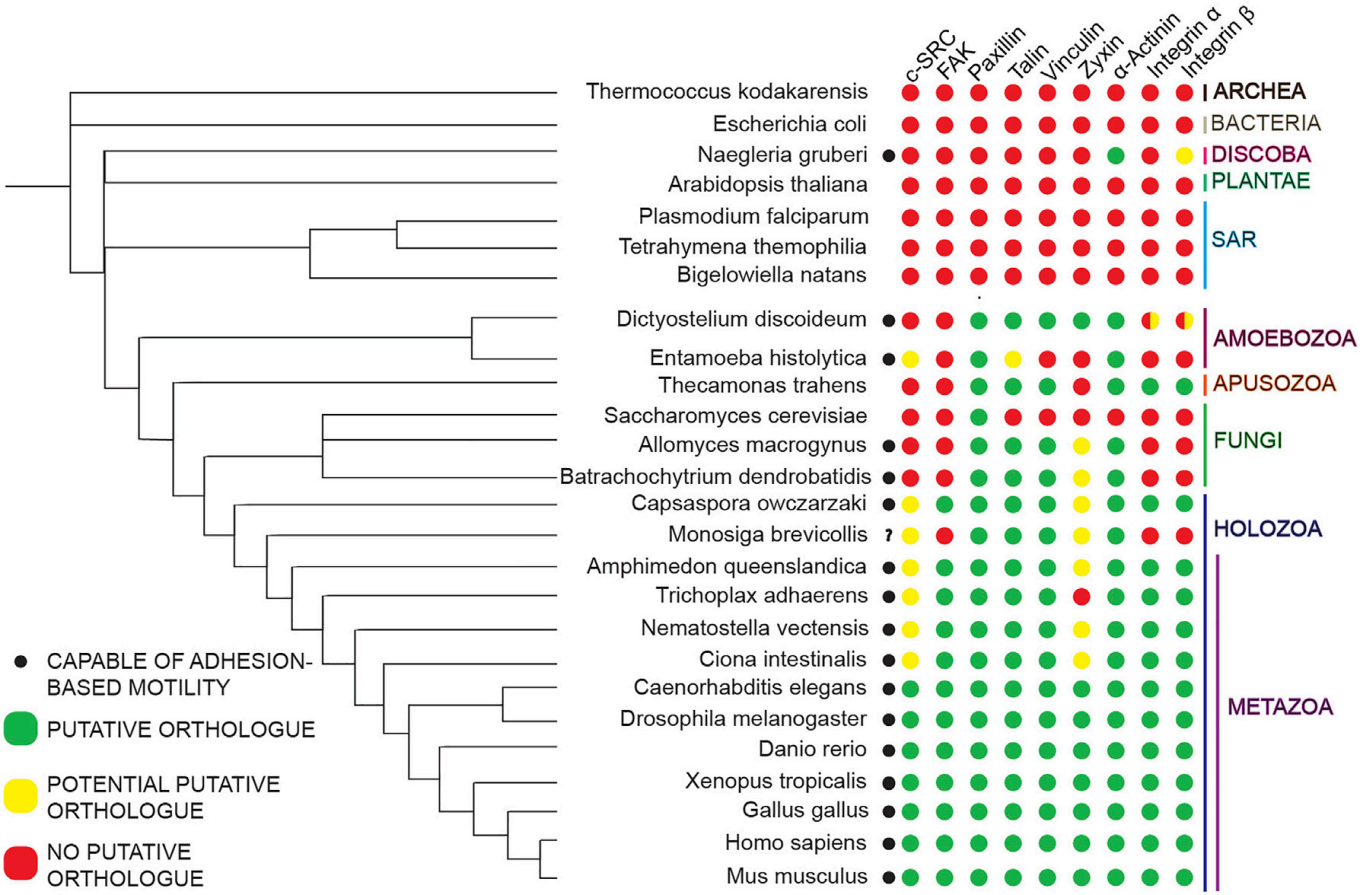
Non-metazoan organisms possess homologues of some but not all core Metazoan focal adhesion components. Bidirectional BLASTP was utilized to map the presence and absence of homologues of core Metazoan focal adhesion components of interest in representative species across evolutionary space. The presence and absence of these homologues, as indicated by the colored dots (legend to the left), is mapped against a species tree derived and expanded from previous work (Velle and Fritz-Laylin, 2019).

In addition to its role in migration, the evolution of integrin-mediated cell-substrate adhesion machinery–such as focal adhesions–is believed to have been critical for independent origins of multicellularity (Hynes and Zhao, 2000; Sebe-Pedros et al., 2010). Though the machinery was originally believed to be Metazoan-specific (Nichols et al., 2006; Rokas, 2008), studies analyzing recently sequenced genomes of organisms distantly related to Metazoans (King et al., 2008; Shalchian-Tabrizi et al., 2008; Ozbek et al., 2010; Sebe-Pedros et al., 2010; Suga et al., 2013) suggest full-length versions of select Metazoan focal adhesion molecules originated just prior to the divergence of the Amoebozoa phylum, with other molecules originating later on (Figure 1). Interestingly, evolutionary analyses of the mechanisms of cell motility across a range of eukaryotic phyla indicate that various organisms lacking putative homologues of select focal adhesion components still utilize adhesion-dependent forms of motility (Fritz-Laylin, 2020), indicating the potential presence of unique adhesomes in organisms evolutionary distant to Metazoa (Figure 2). This potential presence is of particular interest given that understanding how these unique cell-substrate adhesions form, function, and have evolved over Eukaryotic phyla can serve as a cornerstone for further investigating how conserved adhesion-dependent mechanisms such as migration, cytokinesis, cell survival, and other processes have evolved across species.

Investigation into these evolutionary distant cell-substrate adhesions, however, is largely lacking. Like most research in cell biology (Goldstein and King, 2016), the majority of cell-substrate adhesion research focuses on focal adhesions in mammalian cell culture systems, with a small number of studies in Metazoan model organisms such as *Drosophila* and zebrafish. This approach, however, is unamenable to probing evolutionary cell biology questions of how cell-substrate adhesions have evolved across eukaryotic space and what molecules are conserved across Eukaryotic cell-substrate adhesions. As previously discussed (Goldstein and King, 2016), using “non-traditional” model organisms to investigate cell biology mechanisms is advantageous for understanding the evolution and specialization of machinery such as cell-substrate adhesions. Indeed, combining established phylogenomic analyses with cell biology approaches in non-Metazoan model organisms to study evolutionary distant cell-substrate adhesions is an exciting emerging topic within the field of cell adhesions.

Interestingly, while research into non-Metazoan cell-substrate adhesions is a promising novel avenue of investigation, there are gaps within the field of Metazoan focal adhesions that are also emerging as exciting new topics for future work. One such topic is research into *in vivo* focal adhesion formation and dynamics. Currently, most knowledge on focal adhesions has been derived from a wealth of *in vitro* studies in both 2D and 3D mammalian cell culture systems (Cukierman et al., 2001; Yamada et al., 2003; Fraley et al., 2010; Geraldo et al., 2012; Doyle et al., 2015; Doyle and Yamada, 2016; Yamada and Sixt, 2019). Over the past 40 years, *in vitro* models have served as a system for researchers to readily visualize and manipulate focal adhesion structures and dynamics at the cell ventral surface. While this work has provided the foundation for our knowledge of the mechanisms of focal adhesion biology, it is critical to understand how focal adhesion structures are formed and are dynamically regulated in a native *in vivo* environment where signals, mechanics, and environment are intact.

Despite the importance and potential impact of this work, research characterizing focal adhesions and their dynamics *in vivo* is limited. Furthermore, while most *in vivo* work in Metazoan systems has focused on the role of focal adhesions in collective cell migration (Lewellyn et al., 2013; Goodwin et al., 2016; Goodwin et al., 2017; Fischer et al., 2019; Gunawan et al., 2019; Olson and Nechiporuk, 2021; Yamaguchi et al., 2022), research looking into the role of focal adhesions during single-cell migration *in vivo* is limited to one study (Barros-Becker et al., 2017). Further investigation into focal adhesion dynamics and formation *in vivo* is critical for understanding how focal adhesions function in an intact, complex microenvironment compared to a simplified *in vitro* system.

Taken together, novel research into *in vivo* focal adhesions and cell-substrate adhesions in organisms evolutionarily distant from Metazoa–as well as the individual molecules composing these structures–provides exciting new topics of investigation within the field of adhesion-mediated cell migration. In this review, we will highlight work that has been published in these emerging topics with an emphasis on work that directly visualizes cell-substrate adhesions, as well as further discuss the importance and potential implications of further research in these fields. By doing so, we hope to emphasize the potential to use these novel research avenues to further our understanding of both the evolution of cell-substrate adhesion machinery and its utility in complex *in vivo* microenvironments.

## Cell-substrate adhesions in organisms evolutionarily distant to Metazoans

Genome sequencing of non-Metazoan organisms paired with phylogenetic analyses of the presence and absence of homologues of core focal adhesion proteins across Eukaryotic phyla (King et al., 2008; Shalchian-Tabrizi et al., 2008; Ozbek et al., 2010; Sebe-Pedros et al., 2010; Suga et al., 2013), has shown certain core molecules of this cell-substrate machinery originated prior to the divergence of Metazoa. Specifically, mapping of the presence and absence of homologues of full-length versions of core focal adhesion proteins suggests full-length versions of select Metazoan focal adhesion molecules, mostly scaffolding proteins, originated just prior to the divergence of the Amoebozoa phylum, with other molecules originating further along (Figure 1). Consistent with this idea, various non-Metazoan organisms that exist in both unicellular and multicellular states contain putative homologues of only select core focal adhesion proteins, primarily scaffolding molecules, while lacking homologues of other key molecules such as integrins or the tyrosine kinases, Src and focal adhesion kinase (referred to as FAK moving forward) (Figure 2).

Previous research (Fritz-Laylin, 2020) has shown that many non-Metazoans possessing homologues of only a few core focal adhesion components are capable of adhesion-based motility (Figure 2). These results suggest that non-Metazoan organisms are capable of adhesion-based motility despite lacking putative homologues of focal adhesion components shown to be important for Metazoan adhesion formation, indicative of unique adhesomes in these species. Due to the sheer number of proteins identified in Metazoan focal adhesions over the years (Lo, 2006; Kanchanawong et al., 2010; Kuo et al., 2011; Case and Waterman, 2015), many of which have specialized functions at focal adhesion sites, it is likely non-Metazoan adhesomes are composed of a mixture of known homologues of core focal adhesion components and other cell-substrate adhesion molecules unique to the organism in question. Given this scenario, identifying the individual components associated with cell-substrate adhesions in organisms evolutionary distant from Metazoa–as well as the functions, dynamics, and interactions of each molecule at these adhesion sites–is a daunting challenge. Furthermore, due to the various genetic, molecular, and biochemical limitations scientists encounter when working with emerging non-Metazoan systems, cell biological research of cell-substrate adhesion machinery in organisms evolutionary distant from Metazoa is limited.

Despite these limitations, some initial work characterizing cell-substrate adhesions and some of their individual components in non-Metazoan organisms provides a solid foundation for this emerging field moving forward. Here we review some of the recent cell biological work investigating and visualizing cell-substrate adhesions and their individual molecules in non-Metazoan organisms as well as discuss some of the conundrums researchers must consider moving forward.

### Dictyostelium

One non-Metazoan organism of particular interest for studying evolutionarily distant cell-substrate adhesions is the model Amoebozoan, *Dictyostelium discoideum* *. Dictyostelium* is a soil-dwelling social amoeba that exists in both a unicellular and multicellular state and has been extensively utilized to study mechanisms of host-pathogen interactions, development, motility, and cell adhesion (Bozzaro, 2019). It also serves as an intriguing model organism for studying evolutionary distant adhesions due to its use of mechanosensitive adhesions to promote motility in a fashion akin to mammalian-relevant cells such as neutrophils, which predominantly use non-adhesion based migration, but can take advantage of adhesion-based migration mechanisms within specific contexts such as transmigration (Copos et al., 2017).

The nature, composition, and function of these *Dictyostelium* adhesions, however, are largely uncharacterized and somewhat contentious. Previous research (Sebe-Pedros et al., 2010) indicate *Dictyostelium* lacks putative homologues of various components that are pivotal for focal adhesion formation in Metazoans such as integrins and the tyrosine kinases FAK and c-SRC, as well as other core focal adhesion components (Figure 1). Furthermore, *Dictyostelium* also lacks putative homologues of well-established ECM components such as collagen, laminin, and fibronectin (Hynes, 2012). Largely due to the lack of these components, there is some question as to whether a cell-substrate adhesion complex akin to Metazoan focal adhesions exists in *Dictyostelium* (Loomis et al., 2012; Tarantola et al., 2014). Despite lacking these components, however, current research suggests *Dictyostelium* form cell-substrate adhesions using both known focal adhesion component homologues it does possess and molecules unique to the organism.

Like its Metazoan counterparts, *Dictyostelium* possesses two Talin molecules, *talA* and *talB* (Tsujioka et al., 2008). In Metazoans, Talin acts as the mechanosensitive hub of adhesions and serves as the initial physical linker between integrin molecules and the actin cytoskeleton (Klapholz and Brown, 2017; Goult et al., 2018). In *Dictyostelium*, TalA is implicated in cell-substrate adhesion and cytokinesis in the unicellular vegetative state, while TalB is involved in cell motility, force transmission, and development during the transition to a multicellular state (Niewohner et al., 1997; Tsujioka et al., 2004; Tsujioka et al., 2008). Furthermore, both molecules have also been shown to bind to actin and possess conserved FERM and I/LWEQ domains similar to their Metazoan counterparts (Tsujioka et al., 2008; Tsujioka et al., 2019). Intriguingly, double knockouts of *talA* and *talB* in *Dictyostelium* demonstrated a severe defect in cell-substrate adhesion and migration (Tsujioka et al., 2008), suggesting a critical role for both TalA and TalB in both cellular processes.

*Dictyostelium* also possesses homologues of Talin’s cognate binding partner Vinculin; in Metazoan focal adhesions, Vinculin binds Talin to form a molecular clutch pivotal for mechanotransduction and focal adhesion maturation (Klapholz and Brown, 2017; Goult et al., 2018). Interestingly, *Dictyostelium* possesses two putative Vinculin homologues: VinculinA (VinA) and VinculinB (VinB). VinA is associated with cytokinesis B and has been shown to localize to ventral surface punctae (Nagasaki et al., 2009). VinB, meanwhile, is associated with DdEGFL (a synthetic epidermal growth factor-like peptide) enhanced cell migration and has been shown to be in complex with both Talin and actin in *Dictyostelium* (Huber and O'Day, 2012). These results are suggestive of an adhesion complex similar to focal adhesions, though direct ventral surface co-localization of VinB with either Talin or actin has not been confirmed. Furthermore, conservation of a Talin-Vinculin binding axis in *Dictyostelium* has not been confirmed.

Another core focal adhesion molecule conserved in *Dictyostelium* is the scaffolding molecule Paxillin. In Metazoans, this molecule is primarily associated with focal adhesion signal transduction and assembly/disassembly dynamics (Lopez-Colome et al., 2017). The *Dictyostelium* homologue PaxillinB (PaxB) has been shown to localize to ventral surface punctae (Nagasaki et al., 2009), and is involved in actin-centric based processes including cell-substrate adhesion (Bukharova et al., 2005; Pribic et al., 2011). Double knockout of *talA/B* ablates GFP-PaxB ventral surface punctae formation in *Dictyostelium* (Tsujioka et al., 2008), suggestive of a relationship between these proteins at cell-substrate adhesion sites. Interestingly, *Dictyostelium* does not possess a putative homologue of the most well-known Paxillin interactor, FAK, a tyrosine kinase that phosphorylates Paxillin to regulate assembly dynamics (Lopez-Colome et al., 2017) (Figure 2). Furthermore, analysis of the *Dictyostelium* kinome reveals a lack of any putative tyrosine kinases in the model Amoebozoa (Goldberg et al., 2006), suggesting PaxB may not be involved in regulating cell-substrate dynamics *via* tyrosine phosphorylation.

In addition to putative homologues of core focal adhesion proteins, *Dictyostelium* also possesses cell-substrate adhesion molecules unique to the organism. SadA, a novel putative nine-transmembrane protein with conserved EGF domains similar to those in Metazoan β-integrins, is required for cell-substrate adhesion and actin cytoskeleton organization (Fey et al., 2002). Another nine-transmembrane protein regulating cell-substrate adhesion in *Dictyostelium* is Phg1, an orthologue of the human transmembrane protein TM9S4 (Cornillon et al., 2000). Though the exact role of these molecules in *Dictyostelium* cell-substrate adhesion has not been fully elucidated, initial research suggests that these molecules are involved in cell-substrate adhesion *via* regulation of the ventral surface expression and stability of the Amoebozoan-specific adhesion receptor SibA (Froquet et al., 2012).

SibA is a molecule of particular interest when investigating *Dictyostelium* cell-substrate adhesions given its unique profile. Part of the Similar to Integrin β (SIB) family, SibA is expressed at the cell ventral surface and possesses multiple extracellular domains associated with cell-substrate adhesions in Metazoan β-integrins such as a von Willebrand factor type A (VWA) domain and cysteine rich domain (Cornillon et al., 2006). Intriguingly, SibA also contains two conserved NPXY motifs–which integrins use to bind to Talin in Metazoan systems–in its intracellular C-terminus and has been shown to bind *Dictyostelium* Talin molecules *via* this motif (Cornillon et al., 2006). Furthermore, both SibA and its family member SibC have been shown to function redundantly in cell-substrate adhesion (Cornillon et al., 2008).

Taken altogether, the data collected in *Dictyostelium* suggest the presence of a unique cell-substrate adhesion composed of both Amoebozoan-specific molecules and homologues of known Metazoan cell-substrate adhesion proteins (Figure 2). While some of the initial interaction relationships between a few of these proteins have been demonstrated, further work is needed to elucidate the relationships, functions, and dynamics of these molecules at *Dictyostelium* cell-substrate adhesion sites.

### Capsaspora^1^

Part of the Filasterea class and one of the closest unicellular relatives to Metazoa (Suga et al., 2013) capable of transitioning to a multicellular form, *Capsaspora owczarzaki* is uniquely placed phylogenetically (Figure 1) to investigate how cell-substrate machinery has been co-opted for multicellularity (Parra-Acero et al., 2018). Additionally, research focused on the unicellular adhesive stage of the *Capsaspora* lifecycle (Parra-Acero et al., 2020) has shown the utilization of homologues of known Metazoan focal adhesion molecules to promote *Capsaspora* cell-substrate adhesions.

Initial work using immunostaining with antibodies for various homologues of known Metazoan focal adhesion components (specifically, Paxillin, Talin, integrin β2 and Vinculin) demonstrated that these proteins form distinct cell ventral surface punctae that co-localize with actin at filopodia (Parra-Acero, 2019; Parra-Acero et al., 2020). Additionally, integrin β2 was shown to co-localize with both vinculin and talin at some, but not all, punctae at filopodia (Parra-Acero, 2019; Parra-Acero et al., 2020). Interestingly, immunostaining of *Caspaspora* cultures plated on fibronectin-coated plates showed an increase in both *Capsaspora* cell-substrate adhesions and presence of the aforementioned protein homologues at filopodia (Parra-Acero, 2019; Parra-Acero et al., 2020). Blocking the integrin β2-ligand interaction, meanwhile, resulted in decreased *Capsaspora* cell-substrate adhesion (Parra-Acero et al., 2020, Parra-Acero, 2019). Further work using codon-optimized forms of *Capsaspora* integrin β2 have shown these proteins can bind to talin molecules using highly conserved NPXY motifs (Baade et al., 2019).

In addition to the work done characterizing the role of *Capsaspora* integrin and scaffolding molecules for cell-substrate adhesion, further work suggests a potential role for tyrosine kinases at cell-substrate adhesion sites. In Metazoan focal adhesions, tyrosine kinases such as Src and FAK are pivotal for regulating focal adhesion formation and assembly dynamics as well as cell migration (Li et al., 2002; Parsons, 2003; Case and Waterman, 2015). Interestingly, genomic analyses across Eukaryotes suggest these tyrosine kinases appear to be Holozoan-specific molecules (Sebe-Pedros et al., 2010) and coincided with the independent transition to multicellularity within this lineage (Miller, 2012). Analysis of the *Capsaspora* genome identified two putative Src homologues, *CoSrc1* and *CoSrc2* (Schultheiss et al., 2012). These molecules exhibit a high degree of sequence and domain conservation when aligned with mammalian Src proteins and localize to distinct punctae at filopodia in a manner akin to other *Capsaspora* homologues of known Metazoan focal adhesion molecules (Schultheiss et al., 2012).

These cell biological data, combined with *Capsaspora*’s position as the closest unicellular relative to Metazoa and genomic analysis suggesting it has a cell-substrate adhesion toolkit akin to Metazoa (Sebe-Pedros et al., 2010), indicate that *Capsaspora* is a promising organism for characterizing non-Metazoan cell-substrate adhesions. Further investigation into *Capsaspora* cell-substrate adhesions could provide critical insight into the potential role of this machinery for Metazoan multicellularity and how cell-substrate adhesions have evolved across Eukaryota.

### Things to consider moving forward

The emergence of the field of cell-substrate adhesion machinery in organisms evolutionarily distant from Metazoa is a promising avenue of research that can help provide insight into how cell-substrate adhesion machinery and its individual molecules have evolved across evolutionary space. This, in turn, will help dissect the core components and interactions necessary for cell-substrate adhesion formation and function. Researching cell-substrate adhesions through this evolutionary cell biology lens is central to expanding our understanding of highly conserved molecules and mechanisms that serve as the cornerstone for highly conserved adhesion-mediated processes such as migration, cytokinesis, and cell survival.

With this in mind, however, there are multiple nuances that must be considered when investigating cell-substrate adhesion machinery and components in organisms evolutionary distant from Metazoa. Most comparative genomics work across Eukaryotes has focused on the presence and absence of homologous genes, which requires a well-annotated genome to ensure accurate homology calls across organisms. When dealing with organisms evolutionarily distant from Metazoa, however, the lack of fully annotated genomes compared to Metazoan systems can make it difficult to accurately identify cell-substrate adhesion component homologues. Incorporating synteny analysis—which looks at the conservation of gene blocks across species—can serve as a powerful approach to help improve the annotation quality of genomes of organisms evolutionarily distant from Metazoa (Dong et al., 2009; Cheng et al., 2012). By improving annotation quality of genomes from evolutionarily distant organisms, tools such as comparative genomics will become more powerful for identifying putative homologues of core cell-substrate adhesion components. Furthermore, an increased amount of fully annotated genomes can subsequently be utilized to conduct synteny analysis of cell-substrate adhesion genes across Eukaryotic phyla, which has largely been lacking outside a few select studies (Cheng et al., 2012; Zhao and Schranz, 2019). Combining synteny analysis with comparative genomics can thus help paint a more complete picture of the evolution of cell-substrate adhesion machinery across Eukaryotic space.

Building on this, while genomic sequencing across Eukaryotes has helped map out the presence and absence of homologues of core Metazoan focal adhesion molecules (Sebe-Pedros et al., 2010) **(** Figures 1, 2), using popular methods to do this such as structural or sequence homology *via* bidirectional BLAST with full protein sequences is far from perfect. A prime example of this is the aforementioned Sib proteins; bidirectional BLASTP of Metazoan β-integrin molecules does not identify the Amoebozoan Sib molecules as putative homologues off of sequence alone (Sebe-Pedros et al., 2010). Characterization of the Sib proteins shows that while integrin β cell-substrate adhesion features such as the NPXY motif, VWA domain, and cysteine rich domains are conserved, the Sib proteins also possess extracellular amino acid repeat regions (R1-R4) used by bacteria to bind to substrates (Lally et al., 1999; Cornillon et al., 2006). Given that *Dictyostelium* Sib proteins are found to be the only eukaryotic proteins that contained this bacterial feature (Cornillon et al., 2006), it is likely these features were confounding BLASTP analysis such that Sib proteins were not identified as putative integrin homologues *via* sequence homology alone.

Given this, there is an argument to be made for focusing not solely on sequence homology when evaluating potential homologues of core focal adhesion components in organisms evolutionarily distant from Metazoa. Domain architecture-based homology of predicted proteins in non-Metazoan organisms combined with phylogenetics can serve as a powerful tool to identify candidate molecules that possess conserved domain architectures relevant to cell-substrate adhesions. Furthermore, thorough analysis using domain architecture-based homology across Eukaryota can help tease out the emergence and conservation of domain-specific functions for individual molecules, which will go a long way for understanding how specific cell-substrate adhesions molecules have evolved across Eukaryotic phyla (Yang and Bourne, 2009).

Looking at the domains and motifs of proteins such as the Sib proteins can provide insight into both conserved and specialized functions or interactions across evolutionary space. The presence of conserved NPXY and GxxxG motifs found in the cytosolic and transmembrane domains of Sib molecules, respectively, are ideal examples of conserved cell-substrate adhesion-related domains when comparing integrin β and Sib molecules (Cornillon et al., 2006; Cornillon et al., 2008). Interestingly, to the best of our knowledge, *Dictyostelium* does not express ECM proteins possessing an RGD motif during the highly migratory unicellular state; this RGD motif is of particular interest since it is recognized by integrin molecules for cell-substrate adhesion in Metazoan organisms (Yamada and Geiger, 1997). Furthermore, genomic analyses across Eukaryota suggest ECM proteins that Integrins usually bind to in Metazoa such as fibronectin, collagen, and laminin appeared after the divergence of Holozoans (Ozbek et al., 2010; Hynes, 2012). The presence of the aforementioned R1-R4 amino acid repeat regions, however, could be an example of a specialized domain specific to Sib molecules that was subsequently lost in lineages that diverged later. In bacteria, these domains are found in bacterial surface and secreted proteins that mediate adhesion to both substrate and target cells (Lally et al., 1999; Savolainen et al., 2001). Given these data, it is plausible that the R1-R4 repeats are a *Dictyostelium* unique domain necessary for adhesion engagement to a non ECM-protein substrate *via* Sib molecules, while the RGD-motif Integrin-mediated binding to an ECM protein is an innovation specialized after the divergence of Holozoans.

The combination of functional data with domain-specific homology analyses is a necessary step toward answering challenging conundrums. An example of this combination is in investigating the diverse Src network and regulatory systems found in the *Monosiga* choanoflagellate genus. The phylogenetic positioning of choanoflagellates as one of the closest unicellular relatives to Metazoa makes this phylum one of particular interest for investigating the evolution of cell-substrate adhesion machinery (King et al., 2008). Genomic analyses of *Monosiga brevicollis* suggests the choanoflagellate possesses a vast tyrosine kinase network, including homologues of Src subgroup kinases (Manning et al., 2008). Meanwhile, initial functional analysis of the *Monosiga ovata* Src homologue in a murine cell line and fibroblasts showed partial functional conservation of its C-terminal Src kinase (Csk)-mediated regulatory system (Segawa et al., 2006). Expanding this initial knowledge with functional assays centered around the role of these Src molecules in adhesion capabilities will help elucidate how a mysterious phylum such as choanoflagellates utilizes its cell-substrate adhesion machinery.

Another area that will benefit from a combination of functional data and domain-specific homology is the characterization of cell-substrate adhesion interactomes in organisms evolutionarily distant from Metazoa. In Metazoan focal adhesions, interactions and modifications between proteins are pivotal for adhesion function, dynamics, maturation and other processes (Lo, 2006; Kanchanawong et al., 2010; Kuo et al., 2011; Case and Waterman, 2015). It is largely unknown, however, if many of these interactions are conserved in organisms evolutionarily distant from Metazoans. Interactions such as the tyrosine phosphorylation of Paxillin by FAK, which is implicated in regulating focal adhesion turnover in Metazoa (Lopez-Colome et al., 2017), is unlikely to occur in many organisms evolutionarily distant from Metazoa since FAK is a Holozoan-specific innovation based on previous research (Figures 1, 2). While this does not rule out other phosphorylation mechanisms such as serine or threonine phosphorylation being utilized to regulate adhesion turnover in organism evolutionarily distant from Metazoa, the lack of functional data to test these hypotheses leaves this question unanswered.

Furthermore, the scarcity of research towards cell-substrate adhesions in evolutionarily distant organisms also leaves unanswered questions regarding critical interactions between conserved components. A key example is the Talin-Vinculin binding axis, which is pivotal for mechanotransduction in Metazoan focal adhesions (Klapholz and Brown, 2017; Goult et al., 2018). Multiple sequence analyses of Talin and Vinculin molecules across Eukaryotes demonstrate predicted binding interaction sites show a mixed degree of sequence conservation across Eukaryotic space, with some homologues possessing a higher degree of conservation than others. Given this ambiguity, it is unclear if the Talin-Vinculin binding axis is an innovation that evolved at a specific point along evolutionary space with a conserved binding interface or if the binding interaction itself is conserved but occurs through different binding interfaces specific to the organism and homologues in question. Combining functional tools such as microscopy, genetics, and biochemistry with domain-based homology will help resolve some of these unanswered interactomics questions, which in turn will provide a more accurate picture of how cell-substrate adhesions and their individual components have evolved.

Thinking along this perspective also means future research in this field will have to keep a close eye on what established models and knowledge are being used to build hypotheses and how this approach may bias assumptions made when looking at cell-substrate adhesion machinery in organisms evolutionarily distant from Metazoa. Since most of the work on cell-substrate adhesion machinery has focused on Metazoan focal adhesions, researchers may bias towards using the Metazoan focal adhesion as the paradigm of what a “bona fide” cell-substrate adhesion should look like without considering the fact that cell-substrate adhesions in organisms evolutionarily distant from Metazoa are likely specialized for the needs of the organism in question. The presence and absence of clear putative homologues of certain components such as FAK and Src in phyla capable of using adhesion-based motility, such as Amoebozoa, suggest that these phyla likely possess cell-substrate adhesion machinery specialized to the organism.

Following this line of thinking, the role of a specific ECM as a pivotal component of cell-substrate adhesions is another nuance that will need to be carefully considered. Though ECM proteins such as fibronectin, collagen and laminin, among others, are adhered to in Metazoan systems (Case and Waterman, 2015), this is likely not the case in organisms evolutionarily distant from Metazoa. Previous research suggests various ECM proteins such as laminin subunits and fibrillar collagens originated just prior to the divergence of Metazoa, with basement membrane proteins originating just prior to Eumetazoa (Hynes, 2012). While comparative genomics of organisms evolutionarily distant from Metazoa suggests many possess proteins that contain domains characteristic of Metazoan ECM proteins (Ozbek et al., 2010; Sebe-Pedros et al., 2010; Hynes, 2012; Linden and King, 2022) there appears to be a lack of putative ECM protein homologues that resemble Metazoan ECM proteins, suggesting ECM proteins associated with Metazoan focal adhesions are a specialized innovation specific to cell-substrate adhesions in Metazoan systems. This is not to suggest, however, that there are no ECM-like proteins for cell-substrate adhesions in organisms evolutionary distant from Metazoa; it is wholly plausible that some of the ECM protein-like domains found in organisms evolutionarily distant from Metazoa could be associated with cell-substrate adhesions. Additionally, organisms evolutionarily distant from Metazoa may utilize substrate proteins for cell-substrate adhesions that are unique from Metazoan ECM proteins or utilize cell-substrate adhesions that act in a more promiscuous fashion with no specific substrate. If this is the case, it can be speculated the function, composition, and dynamics of evolutionarily distant cell-substrate adhesions may also differ depending on the environment or matrix that organisms utilize or navigate. Further functional analysis of candidate ECM proteins or substrates for cell-substrate adhesion in organisms evolutionarily distant from Metazoa is pivotal to understand if substrates specialized for cell adhesion are utilized by said organisms or are an innovation specific to Metazoa.

It is also key for researchers in this emerging field to not assume that the Metazoan focal adhesion model is the paradigm for “complex” cell-substrate adhesion machinery. It is possible that cell-substrate adhesion machineries in organisms evolutionarily distant from Metazoa are as complex or intricate as the Metazoan counterparts in the number of molecules and binding interactions. The tyrosine kinase signaling network in *Monosiga brevicollis*, which is larger than any Metazoan network (Manning et al., 2008), is a great example of a non-Metazoan organism possessing machinery arguably more diverse and complex than its Metazoan counterparts. With this in mind, research into the field of evolutionarily distant cell-substrate adhesion biology will benefit from unbiased screens such as immunoprecipitation mass spectrometry to identify novel components unique to organisms. Doing so will provide a more complete picture of molecules, both homologues of known cell-substrate adhesion components as well as organism-specific proteins, involved in cell-substrate adhesion machinery in organisms evolutionarily distant from Metazoa. These data can then be applied to more fully understand how cell-substrate adhesion machinery, and the various processes associated with it, have evolved across evolutionary space.

## *In vivo* analysis of focal adhesions

While most of the research on cell-substrate adhesions has been centered around the paradigm of focal adhesions in Metazoa, there are still plenty of unanswered questions and avenues within this sphere. Indeed, though research into cell-substrate adhesion machinery in organisms evolutionarily distant from Metazoa is an exciting new field, there are various characteristics of Metazoan focal adhesions that still need to be elucidated. One such example of one of these potential avenues of interest is research into the characterization of focal adhesions in an *in vivo* setting. The majority of work on Metazoan focal adhesions over the last few decades has been done *in vitro* cell culture models. Though this research has provided key mechanistic insights into focal adhesion biology, *in vitro* models inevitably fail to replicate the complex network of signals, environment, and other factors that influence cellular process such as adhesion formation and migration in an *in vivo* model. Indeed, initial research looking at focal adhesions *in vivo* suggests these structures function differently than those *in vitro* models. Furthermore, although proteomic studies *in vitro* have revealed focal adhesion complexes contain hundreds of proteins (Kuo et al., 2011), only a few select proteins have been characterized *in* *vivo*. Here, we summarize studies investigating direct visualization of focal adhesion components *via* high-resolution imaging approaches during *in vivo* cell migration, focusing on examples of collective and single cell migration.

### Focal adhesion structures during collective cell migration

Focal adhesions have been primarily characterized in collective cell migration during tissue morphogenesis *in vivo*. Although the vast majority of these *in vivo* studies focus on utilizing genetic manipulation or knockdown approaches to test the function of individual focal adhesion components in adhesion formation and function (Dray et al., 2013; Qiao et al., 2014; Haage et al., 2018), some work in the literature has focused on visualizing focal adhesion structures during key cellular processes such as collective cell migration, particularly in the fruit fly, *Drosophila melanogaster,* and the zebrafish, *Danio rerio*.

**FIGURE 3:**
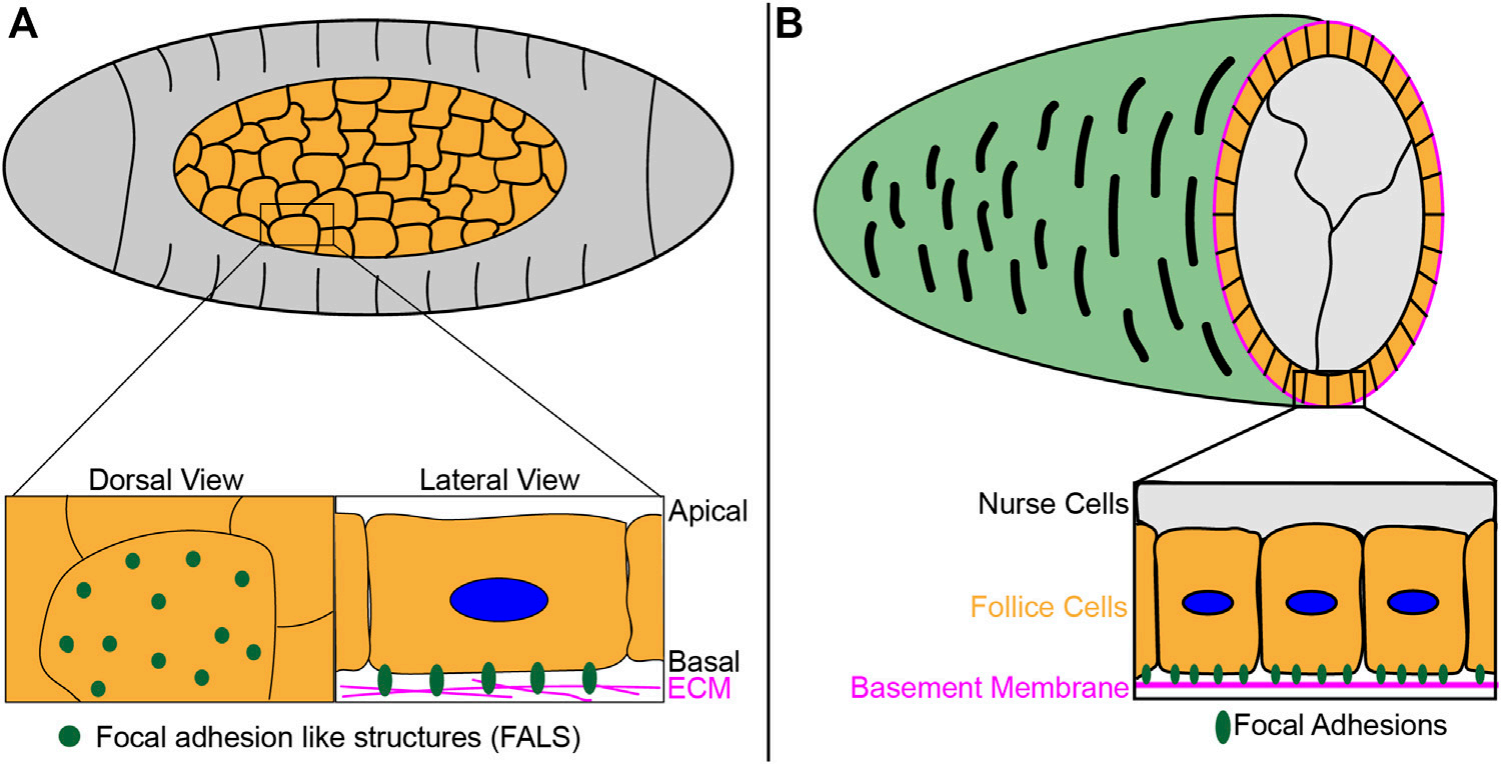
*Drosophila* developmental processes utilize adhesion structures for collective cell migration. **(A)** During *Drosophila* dorsal closure, focal adhesion like sructures (FALS) form at contact sites between the amnioserosa (orange) and the underlying ECM, as epidermal cells (gray) migrate as a sheet and seal on the dorsal side. These structures are required to transmit the necessary tension generated for dorsal closure. For a live imaging example of this process, please reference (Goodwin et al., 2016). **(B)** As follicle cells (orange) collectively migrate during *Drosophila* egg chamber elongation, cells form focal adhesions at contact sites with the underlying ECM-rich basement membrane. For a live imaging example of this process, please reference (Lewellyn et al., 2013).

One such example of this work centers on the role of focal adhesions in *Drosophila melanogaster* amnoiserosa, an extraembryonic tissue involved in several morphogenetic events during embryogenesis (Lacy and Hutson, 2016). Recent work showed the formation of focal adhesion-like structures at amnioserosa-ECM contact sites (Figure 3A), which are required for the transmission of generated tension required for dorsal closure, a process that leads to the sealing of the epidermal sheet of cells on the dorsal side of the embryo (Goodwin et al., 2016; Goodwin et al., 2017). A sheet of epidermal cells migrates over the amnioserosal cells, and the amnioserosal cells contribute to this migration by actively contracting and generating traction forces through interactions with ECM proteins such as laminin, which localizes in between the amnioserosa and yolk contact surface, providing physical environment for focal adhesion formation (Narasimha and Brown, 2004; Goodwin et al., 2016). Focal adhesion components Talin and integrin β localize to these focal adhesion-like structures in amnioserosal cells, with the former shown to be dynamic within these adhesion sites as measured by fluorescence recovery after photobleaching (FRAP). Furthermore, consistent with the presence of Talin and integrin βPS, these structures were shown to be mechanosensitive, increasing in stability and size in response to compression (Goodwin et al., 2017). Unlike the polarized organization and distribution of focal adhesions in migrating cells in *in vitro* culture models, however, the structures seen *in vivo* in amnioserosal cells are evenly distributed, consistent with their non-directional movement during this developmental process (Figure 3A). These results suggest focal adhesion proteins are also dynamically regulated *in vivo* but can function and organize in a mechanism distinct from those seen in cell culture models.

Yet another example of focal adhesion-like structures forming during *Drosophila* development is the involvement of these structures during egg chamber elongation and ellipsoid egg production for oogenesis (Haigo and Bilder, 2011). During the process of elongation, follicular epithelial cells are known to collectively migrate as a sheet along the underlying ECM-rich basement membrane, a prime region for focal adhesion formation made from proteins such as collagen IV, laminin, nidogen and perlacan (Lunstrum et al., 1988; Gutzeit et al., 1991; Horne-Badovinac and Bilder, 2005; Schneider et al., 2006; Haigo and Bilder, 2011; Lerner et al., 2013; Cetera et al., 2014; Horne-Badovinac, 2014) (Figure 3B). Antibody staining of integrin βPS in the follicular epithelium demonstrated enrichment of this component at the follicle cell-basement membrane interface, suggesting cell-matrix interaction and focal adhesion formation are involved in this migration process (Fernandez-Minan et al., 2007). Other focal adhesion components such as integrin heterodimer isoforms (aPS1βPS and aPS2βPS), actin regulators (Zasp and Ena), kinases (FAK and integrin-linked-kinase), and scaffolding hubs (Paxillin, Talin, and Tensin) were also shown to localize to the end of actin filaments at follicle cell-basement membrane contact sites (Bateman et al., 2001; Delon and Brown, 2009). Interestingly, this research also showed that integrin heterodimers at focal adhesions changed from one isoform to another during development, suggesting the function of the adhesion structure also changes throughout the process (Delon and Brown, 2009). Further work looking at follicular epithelial cell migration *via* live imaging, meanwhile, revealed Talin localization at the trailing edge, suggestive of focal adhesion foci formation (Lewellyn et al., 2013). Interestingly, it was also demonstrated that reduced integrin-mediated adhesion promotes collective cell migration, likely due to focal adhesion disassembly at the trailing edge of individual cells (Lewellyn et al., 2013). Taken together, these studies suggest focal adhesion structures at the follicle-ECM surface facilitate follicle cell migration.

**FIGURE 4:**
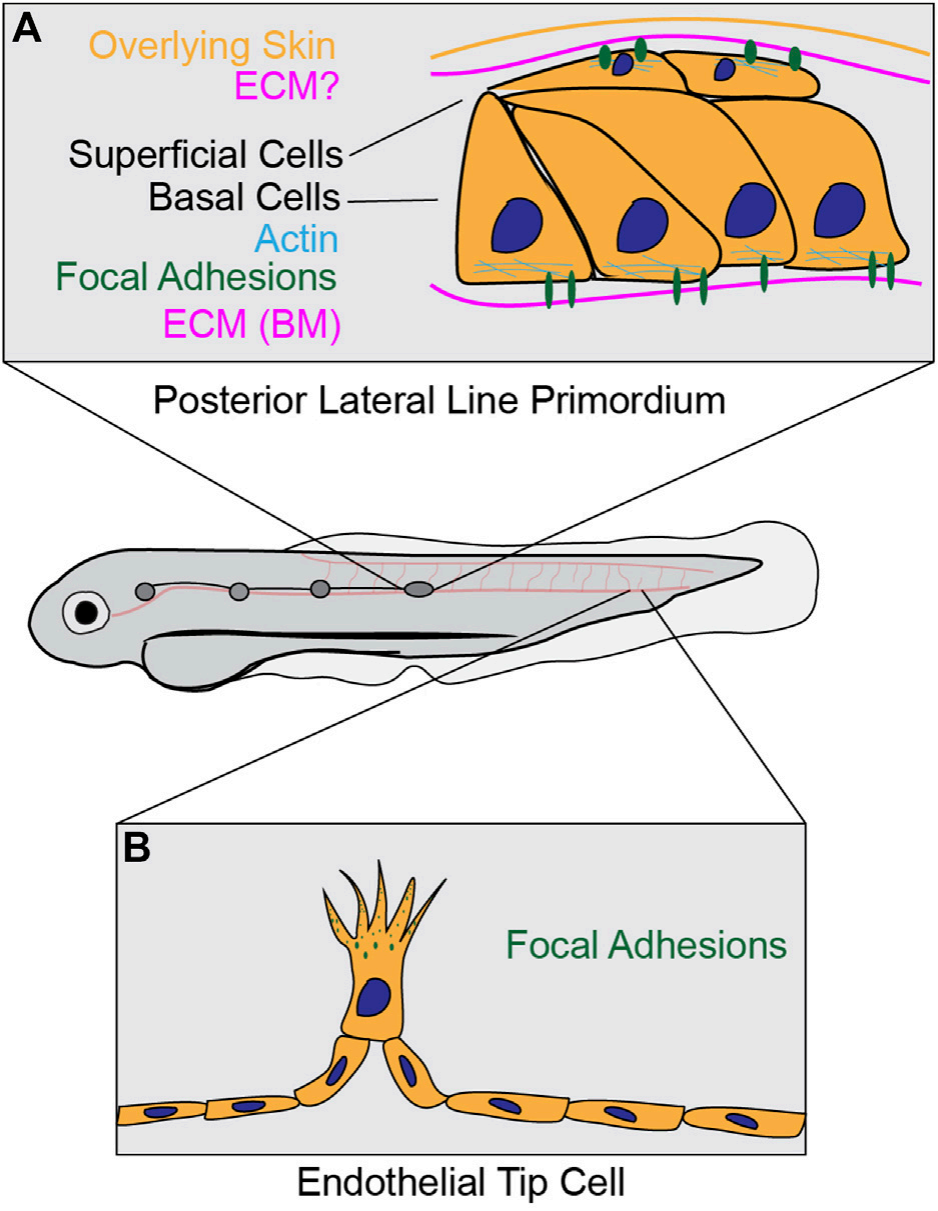
Focal adhesions in zebrafish collective cell migration. **(A)** During pLL primordium migration, basal cells form focal adhesions at contact sites with the ECM at the basement membrane. Additionally, it has been shown that superficial cells form focal adhesions at the apical surface, although it is unknown if there is an ECM layer (question mark) in between superficial cells and the overlying skin. For a live timelapse imaging example of this process, please refer to (Yamaguchi et al., 2022) **(B)** Leader endothelial cells form focal adhesions at cell protrusions during sprouting angiogenesis. After initial formation, adhesion structures stabilize at the distal lamellipodial region before disassembling as the cell continues to migrate onwards. For a live timelapse imaging example of this process, please refer to (Fischer et al., 2019).

In addition to the work done in *Drosophila,* research in the zebrafish, *Danio rerio*, has further elucidated *in vivo* dynamics of focal adhesions. During zebrafish development, the posterior Lateral Line (pLL) primordium is a group of cells known to migrate collectively between the overlying skin and the underlying muscle layer in a coordinated fashion. This migration is required to deposit a cluster of cells that eventually differentiate into mechanosensory organs (Dalle Nogare and Chitnis, 2017). Briefly, cells at the front of the primordium, called “leader” cells, probe the environment *via* focal adhesions at their extending fronts to establish directional migration. Behind these leader cells is a reservoir of “follower” cells that physically interact with surrounding cells *via* adherence junctions and help contribute to primordium directionality, force generation, and efficient migration (Qin et al., 2021). During this process, both the leader and follower cells form close contacts with the surrounding ECM layers and segregate into 2 cell layers: basal cells and superficial cells (Dalle Nogare and Chitnis, 2017; Dalle Nogare et al., 2020) (Figure 4A). The basal cells are in direct contact with the basement membrane, which is enriched of laminin and collagen IV (Yamaguchi et al., 2022), while the superficial cells are in direct contact with the overlaying skin, which is thought to possess an ECM, though its components are unknown. Work investigating this process has demonstrated the overlying ECM in the skin is critical for lamellipodial formation in both superficial and basal cells, indirectly suggesting focal adhesion structures form during the migratory process (Dalle Nogare et al., 2020).

Further work in the pLL primordium using fixed imaging approaches demonstrate that superficial primordium cells form lamellipodial-like protrusions during migration, and activated Paxillin (as measured by phospho-Y118-Paxillin staining) localizes to the interface where superficial primordium cells make contact with the skin, providing evidence that focal adhesions form and are activated during primordium migration (Olson and Nechiporuk, 2021). Live imaging studies of core focal adhesion components show the formation of focal adhesion structures on the basal side of basal cells and apical sides of superficial cells, both of which are sites of contact with the surrounding ECM (Yamaguchi et al., 2022). Basal cell adhesions are enriched for integrin β1b and Talin clusters, which co-localize with F-actin and are required for efficient migration, while apical cell adhesions were shown to only localize integrin β1b (Yamaguchi et al., 2022). Interestingly, the basal adhesion structures were shown to be short-lived and transient, with a reported lifetime of less than 2 minutes; furthermore, these *in vivo* structures are less than 2 μm in size, smaller than the reported size of focal adhesion structures in cell culture models. Taken together, this work suggests that *in vivo* and *in vitro* focal adhesion structures exhibit differential dynamics and properties, which could contribute to potential functional differences.

While the formation of the pLL primordium is a well-studied process for revealing mechanisms of collective cell migration, other forms of collective migration in zebrafish have been shown to also require focal adhesions. Work highlighting collectively migrating endocardial cells during zebrafish heart valve morphogenesis using immunostaining approaches showed that various focal adhesion proteins, including integrin β1, integrin α5, Talin, Vinculin, and activated Paxillin, localize to the leading edge of migrating leader endocardial cells (Gunawan et al., 2019). Focal adhesions have also been shown to form at leader cells during zebrafish sprouting angiogenesis (Figure 4B). Live imaging of the focal adhesion component VASP showed localization to filopodial tips and elongated plaques resembling focal adhesion structures at the distal regions of lamellipodial protrusions of tip cells (Fischer et al., 2019; Figure 4D). These VASP-positive structures were shown to initially form at the filopodia tip before stabilizing at the base of protrusions; as the endothelial cell tip advances, these VASP-positive plaques then disassemble, in a fashion akin to the process of focal adhesion maturation seen in cell culture models (Fischer et al., 2019). Interestingly, the lifetime of these *in vivo* VASP-positive structures is comparable to the lifetime of *in vitro* focal adhesions, suggesting focal adhesion lifetime *in vivo* might be similar to the lifetime that measured *in vitro*. These results suggest the presence of focal adhesion structures in leader cells during several forms of collective cell migration.

Though work in *Drosophila* and zebrafish are a promising starting point for investigating focal adhesion formation *in vivo*, these efforts have not been replicated in mammalian systems. To our knowledge, there are no studies visualizing focal adhesions *via* live intravital imaging in mammalian models, with most work focused on immunostaining of fixed samples instead. For example, antibody staining of mouse lung sections with cancer metastases showed the co-localization of actin and integrin α5 in lung-colonized breast cancer cells (Shibue et al., 2013). Furthermore, these components formed elongated adhesion plaques resembling focal adhesions, suggesting cell-matrix adhesions are involved in cancer metastasis (Shibue et al., 2013).

In summary, thanks to initial work focused on the developmental processes of Metazoan model organisms such as zebrafish and *Drosophila,* it is clear that focal adhesion formation and interaction with the ECM is a pivotal component of *in vivo* collective cell migration. Genomic analyses of model organisms such as *Drosophila* and zebrafish suggest these species possess highly conserved focal adhesion toolkits (Figure 2). This notion, paired with the tools that have been developed to help image *in vivo* processes, make these organisms ideal to research focal adhesion structures in a relevant *in vivo* environment. Initial characterization of focal adhesion components Talin and integrin β has shown that *in vivo* structures positive for these components appear to be smaller in size and possess faster turnover dynamics than their *in vitro* counterparts. Indeed, unlike *in vitro* focal adhesions that can be categorized into newly formed nascent adhesions and more mature adhesions, *in vivo* focal adhesions are largely reminiscent of nascent adhesions, suggesting effective focal adhesion-based cell migration may rely on more transient adhesion formation rather than mature and prolonged cell-matrix interactions.

### Focal adhesion structures during single cell migration

While studies into *in vivo* focal adhesions for collective cell migration are growing, work into the role of focal adhesions during *in vivo* single cell migration is largely lacking. Although there has been work focusing on cell-matrix adhesion during single cells adopting cell shape changes *in vivo* (Hagedorn et al., 2009; Ihara et al., 2011; Matus et al., 2014), to the best of our knowledge, only one study has examined *in vivo* focal adhesion dynamics during single cell migration. This work, using a transgenic zebrafish model, focused on characterizing Paxillin localization in actively migrating single cells (Barros-Becker et al., 2017). Transient expression of fluorescently-tagged Paxillin under a macrophage-specific promoter showed that macrophages form dynamic Paxillin-positive punctae at the mid-front of the migrating cell body (Barros-Becker et al., 2017). These punctae formed at cell protrusion sites and dispersed when the protrusions retracted (Barros-Becker et al., 2017). Work from our own lab using the same-species transplantation of highly migratory zebrafish melanoma cells expressing fluorescently labeled Paxillin showed that Paxillin co-localizes with actin to form dynamic focal adhesion structures at sites of cell-ECM contact during single cell migration (Xue et al., 2022). Taken together, these studies suggest that focal adhesion structures form during *in vivo* single cell migration.

Compared to *in vivo* collective cell migration, research into focal adhesions during *in vivo* single cell migration is still in an early stage. The only conclusions that can be established with current research into focal adhesions during *in vivo* single cell migration is Paxillin is present at adhesion contact sites with the ECM and is dynamically regulated. Similar to the trend seen in collective cell migration, *in vivo* focal adhesions are smaller during single cell migration and have a morphology reminiscent of nascent adhesions in cells under *in vitro* cell culture conditions.

One factor that may limit research is a lack of physiological processes involving *in vivo* single cell migration. Although we anticipate that the function and composition of focal adhesion machinery will not differ drastically between collective and single cell migration *in vivo*, it is still crucial to investigate these questions. Furthermore, most of the mechanistic insight of focal adhesion biology generated *in vitro* has been from studies focused on single cell migration. Given this, focal adhesion-based *in vivo* single cell migration holds promise as serving as a direct way to compare focal adhesion mechanics, formation, and dynamics between an *in vivo* native environment and an *in vitro* cell culture system. Insights gained from this research will go a long way to understand the mechanistic basis of leukocyte and cancer cell migration, which can then be applied for targeting disease-associated processes such as cancer dissemination.

### Things to consider moving forward

Despite this initial work into the role of *in vivo* focal adhesions in cell migration, there are still multiple challenges moving forward. One of the largest obstacles to further research in both *in vivo* collective and single cell migration is the difficulty of imaging cells in a non-invasive fashion while retaining high spatial-temporal resolution. It is important to note that most published *in vivo* studies take advantage of a superficial imaging plane to visualize focal adhesions such as the zebrafish pLL primordium just underneath the skin, and the follicle cell migration at the surface of the *Drosophila* egg. While identification of superficial tissue environments for collective cell migration can provide ideal imaging conditions, this is often not the case for single cell migration. Single cells such as cancer cells and macrophages often migrate in deeper tissue throughout the body, likely introducing ECM variability not as observed in collective cell migration. These observations provide plenty of unanswered questions for researchers to further consider when identifying relevant tissue environments. Will different types of ECM substrate within different microenvironments differentially dictate the formation of focal adhesions? Will focal adhesions adopt differential spatial-temporal dynamics in response to these different environmental substrates? Given the current limitations of non-invasive imaging techniques, it is likely that visualizing focal adhesions during cell migration *in vivo* will continue to require the identification of superficial imaging planes to help answer these questions and many more.

Another challenge associated with *in vivo* focal adhesion research is quantifying protein localization and dynamics at the single cell level, especially in the context of collective migration where cells are physically connected to one another during this process. Given the cell-cell contact during collective cell migration, it can be difficult to distinguish the spatial regulation of focal adhesion structures at single cell resolution, as adhesions localizing at or near membrane contact sites between trailing and leading edges of individual cells become difficult to resolve. A method to potentially overcome this challenge is mosaic labelling of cells, which will reduce fluorescence from neighboring cells and enable better spatial resolution at the single cell scale.

Additionally, single cell migration through a complex tissue or environment is another process where quantifying protein localization and dynamics can present challenges. Though one advantage of single cell migration is the lack of neighboring cells within a collective that can muddle the signal to noise ratio of cells of interest, there are still scenarios such as migration through tissues interfaces where confounding factors could arise. One key example is research looking at the role of focal adhesions in dividing ectodermal cells during macrophage infiltration in *Drosophila*. In this example, it was shown that focal adhesion components localize to punctate structures at the ectodermal-mesodermal tissue interface and that disassembly of these structures enables macrophage infiltration, which was further confirmed *via* RNA interference of focal adhesion components talin, vinculin and integrin β (Akhmanova et al., 2022). This work provides an example of not only the complex environments that single cells likely migrate within but also the possibility that other cells or tissue in the environment may contribute focal adhesions of their own that regulate the ability of cells to migrate within the environment.

As researchers develop techniques to overcome these challenges, future work on *in vivo* focal adhesion structures in both collective and single cell migration is promising. This includes investigating the presence, interactions, and dynamics of individual focal adhesion components, elucidating the overall focal adhesion architecture, and teasing out the spatial organization of these structures in both single cells and collective cell groups.

## Conclusion

Decades after the discovery of focal adhesions and the initial studies characterizing their basic functions and components, (Abercrombie et al., 1971; Geiger, 1979; Burridge and Connell, 1983; Zamir et al., 1999), the field of cell-substrate adhesion research has grown substantially as more researchers build on the wealth of knowledge related to these structures. Despite this wealth, there is still a vast amount of new, specialized avenues of research that will add greater nuances to this topic. Work focused on characterizing cell-substrate adhesions in organisms evolutionarily distant from Metazoa, particularly in concordance with establishing experimental techniques for emerging model organisms, can help elucidate how this machinery has evolved across Eukaryotic space. Insights gained from this research can reveal the core functions and components that are evolutionary conserved, which can subsequently be investigated within the context of other adhesion-dependent cellular processes. Concurrently, further research into focal adhesions *in vivo* can elucidate physiologically relevant dynamics and functions that these structures possess during processes such as migration or proliferation in a physiologically-relevant environment. These insights can then be applied into disease contexts such as cancer with the assurance that physiological nuances and complexities are largely accounted for, providing a more accurate picture when considering potential disease therapies or interventions focused around cell-substrate adhesion machinery.

The advent of these new avenues of investigation, as well as other future directions not covered in this review, are fields full of promise for researchers interested in pushing the boundaries of cell-substrate adhesion machinery. These new findings will help contribute to a more holistic understanding of the field of cell-substrate adhesions machinery, which will go a long way when applying this understanding to other areas of study such as cell migration, proliferation, survival, and pathogenesis.

## Footnotes

1 To the best of our knowledge, there is no established nomenclature for *Capsaspora* genes and proteins. Given this, we followed the nomenclature utilized in Parra-Acero et al., 2020, Schultheiss et al., 2012 and Parra-Acero, 2019.
